# Lord Salisbury and Paying Patients

**Published:** 1912-07-20

**Authors:** Edward A. Attwood

**Affiliations:** Secretary. London Homœopathic Hospital, Great Ormond Street, London, W.C.


### THE EDITOR'S LETTER-BOX.

### Lord Salisbury and Paying Patients.

*To the Editor of* The Hospital.

Sir,—Lord Salisbury suggests that the great hospitals should provide accommodation which would appeal to well-to-do people, who now go into nursing homes when ill.

"He says the great hospitals should exercise more discrimination in their treatment, so that patients who could afford to pay should be made to do so, and great hospitals should provide paying accommodation, so as to attract patients who were not only not poor, but even rich. The other day when the Duchess of Connaught was struck down by illness in Canada she went to a hospital. If that was so in Canada, why not in England? Why should they not here cater for every class beside the poor?"

It may interest your readers to learn that the Board of Management of the London Homœopathic Hospital, Great Ormond Street, W.C., has fitted up, in their new Sir Henry Tyler wing, ten separate bedrooms for the accommodation of paying patients, and they are being much sought after, and evidently fill a "long-felt want."

The private paying rooms have a pleasant outlook on to the ornamental gardens at the corner of Queen Square and Great Ormond Street. They are on one floor, with baths and every modern sanitary convenience. There is a passenger lift from the ground floor. An operating theatre, fitted upon the most approved aseptic principles and situated on the same landing, has been specially built for the private paying patients. These ten separate rooms for paying patients are in addition to the 156 beds for the sick poor in the general wards, for admission to which poverty and sickness are the only qualifications necessary, and for the maintenance of which some £12,000 per annum has to be raised.

Donations towards the Maintenance Fund, or inquiries as to the Paying Wards, should be sent to Yours faithfully,

Edward A. Attwood, Secretary.

London Homœopathic Hospital,
Great Ormond Street, London, W.C.

